# Patient self-referral patterns in a developing country: characteristics, prevalence, and predictors

**DOI:** 10.1186/s12913-024-11115-8

**Published:** 2024-05-21

**Authors:** Mohammad Jahid Hasan, Md. Abdur Rafi, Nahida Hannan Nishat, Ima Islam, Nusrat Afrin, Bikona Ghosh, Etminan Kabir, Samiha Zaman Akhter, Maisha Zaman Poushi, Saadi Abdullah Bin Shahnoor, Jannatul Fardous, Tamanna Tabassum, Sadia Islam, Sumiya Bent Kalam, Mehjabeen Tasnuva Aslam, Taufique Joarder

**Affiliations:** 1 Tropical Disease and Health Research Center, Dhaka, 1100 Bangladesh; 2 Pi Research and Development Center, Dhaka, 1100 Bangladesh; 3 Dhaka Medical College and Hospital, Dhaka, 1100 Bangladesh; 4 SingHealth Duke-NUS Global Health Institute, Singapore, 169857 Singapore; 5 Delta Medical College & Hospital, Dhaka, 1216 Bangladesh

**Keywords:** Referral System, Self-referral, Health System, Tertiary Care hospitals, Bangladesh

## Abstract

**Background:**

Efficient healthcare delivery and access to specialized care rely heavily on a well-established healthcare sector referral system. However, the referral system faces significant challenges in developing nations like Bangladesh. This study aimed to assess self-referral prevalence among patients attending tertiary care hospitals in Bangladesh and identify the associated factors.

**Methods:**

This cross-sectional study was conducted at two tertiary care hospital, involving 822 patients visiting their outpatient or inpatient departments. A semi-structured questionnaire was used for data collection. The patients’ mode of referral (self-referral or institutional referral) was considered the outcome variable.

**Results:**

Approximately 58% of the participants were unaware of the referral system. Of all, 59% (485 out of 822) of patients visiting tertiary care hospitals were self-referred, while 41% were referred by other healthcare facilities. The primary reasons for self-referral were inadequate treatment (28%), inadequate facilities (23%), critical cases (14%), and lack of expert physicians (8%). In contrast, institutional referrals were mainly attributed to inadequate facilities to treat the patient (53%), inadequate treatment (47%), difficult-to-treat cases (44%), and lack of expert physicians (31%) at the time of referral. The private facilities received a higher proportion of self-referred patients compared to government hospitals (68% vs. 56%, *p* < 0.001). Among patients attending the study sites through institutional referral, approximately 10% were referred from community clinics, 6% from union sub-centers, 25% from upazila health complexes, 22% from district hospitals, 22% from other tertiary care hospitals, and 42% from private clinics. Patients visiting the outpatient department (adjusted odds ratio [aOR] 3.3, 95% confidence interval [CI] 2.28–4.82, *p* < 0.001), residing in urban areas (aOR 1.29, 95% CI 1.04–1.64, *p* = 0.007), belonging to middle- and high-income families (aOR 1.34, 95% CI 1.03–1.62, *p* = 0.014, and aOR 1.98, 95% CI 1.54–2.46, *p* = 0.005, respectively), and living within 20 km of healthcare facilities (aOR 3.15, 95% CI 2.24–4.44, *p*-value < 0.001) exhibited a higher tendency for self-referral to tertiary care facilities.

**Conclusions:**

A considerable number of patients in Bangladesh, particularly those from affluent urban areas and proximity to healthcare facilities, tend to self-refer to tertiary care centers. Inadequacy of facilities in primary care centers significantly influences patients to opt for self-referral.

**Supplementary Information:**

The online version contains supplementary material available at 10.1186/s12913-024-11115-8.

## Background

A well-functioning referral system stands as a cornerstone in ensuring optimal patient care within any healthcare ecosystem. It acts as the conduit for primary healthcare providers to connect patients with specialized healthcare resources and services beyond their immediate purview. However, implementing effective referral policies between primary healthcare facilities and higher-level institutions presents significant challenges, particularly in low- and middle-income countries (LMICs) [1].

The major stake in the healthcare of Bangladesh is held by the public sector, which operates on a hierarchical model, where primary care facilities refer patients to higher-level facilities based on the complexity of their medical conditions. Primary healthcare services in this country are typically dispensed through facilities situated at the sub-district levels, which include community clinics, union sub-centers, and upazila health complexes. According to the healthcare delivery model of Bangladesh, there is one community clinic for every 6000 population, which provides only outpatient-based services and some limited medicines by trained community healthcare providers [2]. After community clinics, union health sub-centers also provide outpatient-based services, with a few sub-centers having maternal and childcare services like normal delivery facilities [3]. Above the union sub-centers are upazila health complexes, usually 50-bedded hospitals with inpatient, outpatient, and emergency care facilities. Upazilas are the sub-district level administrative units of the country. The major portion of primary care is provided by these hospitals, which act as the major referring centers for complicated patients. Secondary care services are provided in district hospitals, which provide emergency, inpatient, outpatient, and some specialized care, while tertiary care services are concentrated in medical college hospitals and specialized treatment centers like national institutions of different specialties mostly situated at divisional levels or at the capital city of the country [4, 5].

Ideally, primary healthcare centers should serve as the linchpin in connecting patients with higher-level referral facilities, including secondary and tertiary care hospitals. Patients are supposed to be referred to the higher-level facilities from the primary level with proper referral notes from the attending physicians indicating the medical condition and proper cause of referral. However, reality paints a different picture, wherein the limited resources available in these centers often compel individuals to bypass them and seek healthcare directly from secondary or tertiary facilities [6, 7]. This practice results in the underutilization of most publicly-provided primary care facilities and disrupts the intended linkage within the referral system. Consequently, secondary and tertiary hospitals face an increased patient load, potential waste of resources, and the specialized skills of healthcare providers expected to deliver advanced care. In secondary and tertiary facilities of Bangladesh, the bed occupancy ratio stands at almost 148.2% and 137%, respectively, whereas it remains at only 79% in primary care facilities [8]. This situation compromises the quality of urgent medical attention that referral hospitals are meant to provide due to the overburden of patients [9].

This disparity underscores the possibility of an inefficient referral system in Bangladesh, potentially stemming from patients’ tendencies to self-refer due to potentially less complex medical conditions that could be managed at primary or secondary care facilities. However, there is hardly any evidence regarding the proportion of patients at the tertiary care facilities who are self-referred, bypassing the primary care centers and secondary care centers. Thus, the current study aimed to assess the prevalence and identify factors associated with patients’ self-referral to selected tertiary hospitals in Bangladesh.

## Methods

### Understanding the health system in Bangladesh

**Fig. 1:**
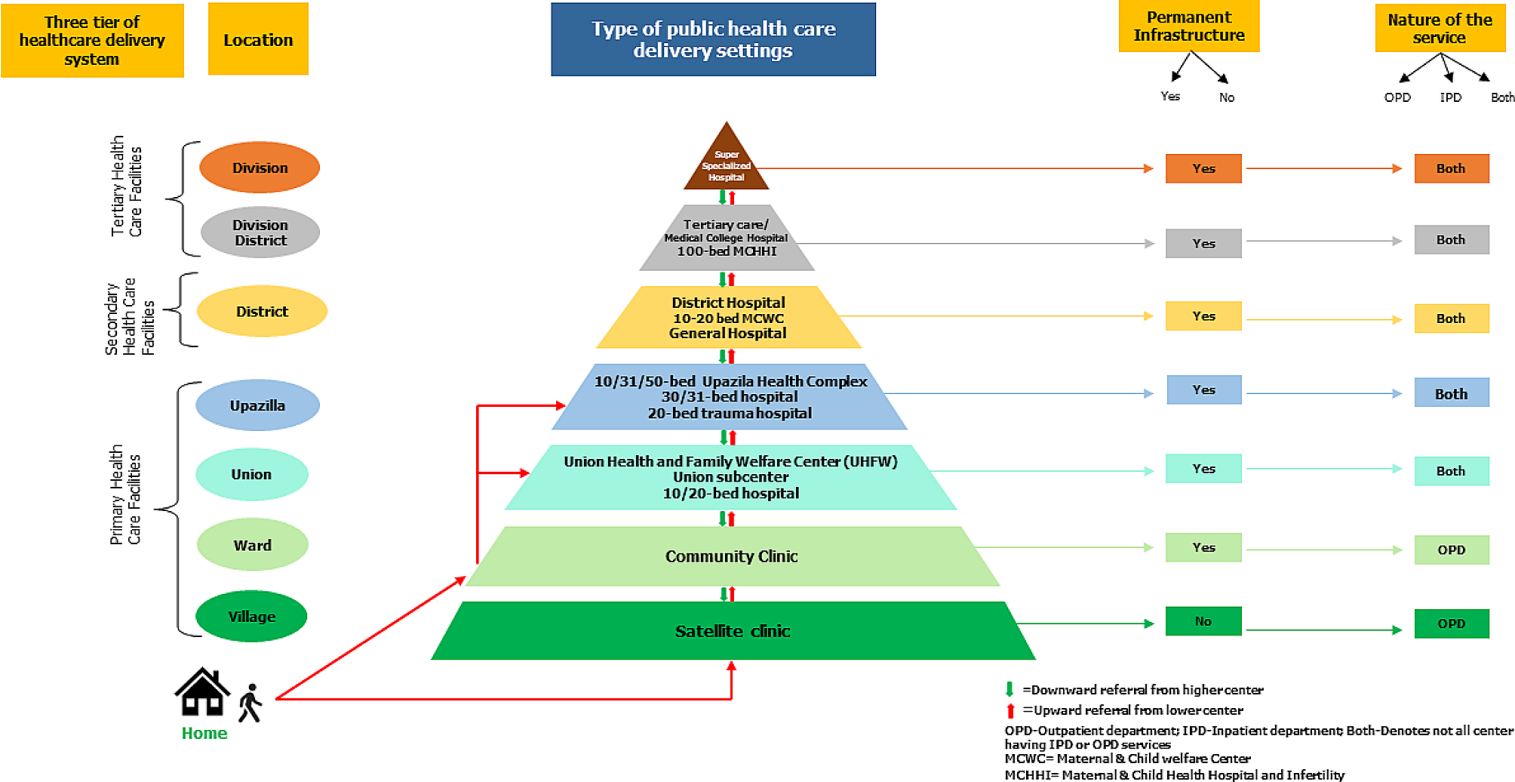
Healthcare delivery system in Bangladesh and referral pathways

Bangladesh has a hierarchical structure of health system across the country (Fig. 1). Satellite Clinics (SC) at the village level and Community Clinics (CC) at ward level offer basic healthcare services with a focus on preventive care and health education for local residents [10–15]. Further up, Union Health and Family Welfare Centers (UHFWC), union sub-centers, and rural health centers provide outpatient services to a larger community at the union level. At the sub-district level, Upazila Health Complexes offer a wide range of healthcare services and act as the first point of contact for health concerns [16]. District Hospitals serve as secondary care centers, offering comprehensive medical care and accepting referrals from primary facilities. These hospitals provide both outpatient and inpatient services, including emergency care [17, 18, 19]. At the pinnacle are tertiary care hospitals, which offer specialized medical services and are equipped with various disciplines and advanced laboratory facilities. Located in divisional headquarters and at national level, these hospitals handle complex medical conditions and provide specialized care not available at lower levels [16, 18]. More detailed description of the health system in Bangladesh are available at Supplementary file 1.

### Study setting

This cross-sectional study was conducted between January and December 2022 at Dhaka Medical College Hospital and Delta Medical College Hospital. Dhaka Medical College Hospital is the largest tertiary-level hospital in Bangladesh, with a 2400-bed facility that caters to both adults and children and handles a substantial caseload annually [20]. With a specialized multidisciplinary team adept at managing a wide array of diseases, this hospital serves as a hub for referrals and admissions from nearby districts and cities. Notably, it also receives referrals from remote areas across the country, particularly for critically ill patients [20, 21].

Delta Medical College Hospital, on the other hand, is a renowned private tertiary care facility in the country, having 350 inpatient beds facility. It offers comprehensive general medical treatment alongside a full spectrum of diagnostic facilities, a surgical department, and specialized units for cancer management. Despite being a private institution, it attends to a significant number of patients seeking treatment for various health issues, both on an outpatient and inpatient basis [22].

### Participants

The study encompassed all individuals who sought medical care in the inpatient and outpatient departments of the designated hospitals during the study period. To determine the sample size, the formula used was n = z^2^p(1-p)/d^2^, where z represents 1.96 for a 95% confidence level, p denotes the estimated proportion of self-referral (anticipated as 50% due to the absence of available data regarding self-referral among Bangladeshi patients), and d signifies the allowable error. With a 95% confidence interval and a 5% error margin, the calculated sample size was estimated at 384. Factoring in an expected 80% response rate and adjusting for a design effect of 1.8 due to potentially high variance of the samples due to hospital size and nature of the hospitals (government vs. private), the total number of required participants was estimated at 864.

The inclusion criteria comprised new adult patients/attendants of any gender seeking medical attention in the outpatient or inpatient departments of Dhaka Medical College Hospital and Delta Medical College Hospital. Exclusion criteria involved returning patients attending for follow-up, individuals seeking services unrelated to medical care (such as immunization or family planning), and critically ill patients admitted to the intensive care unit (ICU) or high dependency unit (HDU). Recruitment of participants from seven key departments—Medicine, Surgery, Gynecology and Obstetrics, Pediatrics, Ophthalmology, Otolaryngology, and Orthopedics—was carried out using these inclusion and exclusion criteria. A systematic random sampling technique was used to recruit the participants. At first, we reviewed the records of the hospitals for the last month and estimated the average number of patients admitted daily in the respective inpatient departments or visiting outpatient departments for the calculation of the sampling interval, which was determined as 11. We included the first patient who was admitted to the respective department or visited the outpatient department after 9 AM, and after that, every twelfth patient was included up to 2 PM if they met the inclusion criteria. Thus, a total of 822 patients were included from both hospitals.

### Data collection

Data collection involved conducting face-to-face interviews either directly with the patients or, in cases where patients were unable to participate (e.g., due to severe illness or being children), with their attendants. These interviews were carried out by trained research assistants, specifically fifth-year medical students or intern doctors from the respective medical colleges involved in clinical attachments. The data collection process utilized a semi-structured questionnaire designed in three sections: Socio-demographic information, gathering details encompassing age, gender, family income, place of residence, and related factors concerning the patients. Medical information: capturing data on presenting complaints, department of admission, specialized medical services required, and similar medically relevant aspects. Referral-related information: Exploring patients’ awareness regarding the referral system (awareness about the referral system was defined from the question, ‘Have you heard about referral system in healthcare? ), the method of referral (whether self-referral or institutional referral), the reasons driving their choice of referral, and related factors influencing their decisions. The questionnaire was developed by an expert panel of researchers, drawing from previous published studies on the topic and their personal work experience in the Bangladeshi health system. Following the initial draft, thorough discussions were held with all co-investigators to refine and incorporate valuable insights into the final version. Subsequently, the questionnaire underwent a pre-testing phase involving 30 patients from Dhaka Medical College Hospital, who were not part of the final analysis. After pre-testing, the questionnaire was amended according to the findings in terms of linguistic clarification. Quality assurance for data collection was carried out by NHN for Dhaka Medical College Hospital (DMCH) and SI for Delta Medical College Hospital. The overall quality assurance process was overseen by the lead author to ensure consistency and accuracy across all data sources.

### Variables

#### Outcome variable

The study’s outcome variable centered on the patients’ mode of referral, categorized into two groups: self-referral and institutional referral. Self-referral was characterized by patients, their families, or acquaintances directly seeking tertiary care without utilizing primary care facilities. Conversely, institutional referral encompassed patients referred by primary, secondary, or other tertiary care facilities. Additionally, the study evaluated the reasons motivating patients to seek referral to tertiary care hospitals.

#### Independent variables

The collected data encompassed patients’ socio-demographic details, including age, gender, family income, place of residence, educational attainment, employment status, and medical information, such as presenting complaints, department of admission, and specialty. The details of the variables can be found in Supplementary file 2.

#### Ethics statement

The study protocol underwent review and received approval from the Ethical Review Committee (ERC) of Dhaka Medical College (ERC-DMC/ECC/2020/78). Prior to participation, all individuals provided informed written consent. In instances where participants could not provide consent themselves, consent was obtained from the attending guardian. The study adheres strictly to the ethical principles outlined in the Declaration of Helsinki.

#### Statistical analysis

All statistical analyses were conducted using Stata version 17.0. Descriptive statistics, such as mean with standard deviation (SD) for continuous variables and frequency with percentage for categorical variables, were employed. Chi-square test was utilized whenver necessary. To ascertain factors associated with patients’ self-referral to the tertiary care hospital, logistic regression models were employed. Initially, a binary logistic regression was performed to identify potential factors correlated with self-referral. Subsequently, a multiple logistic regression model was constructed using the factors that emerged as statistically significant in the binary regression. Thus, hospital type (government or private), department (inpatient or outpatient), residence of the patient (rural or urban), family income of the patients, and distance of hospital from patients’ residence were included in the multiple logistic regression model. Odds ratios (OR) with a 95% confidence interval (CI) were computed. A two-sided *p*-value of < 0.05 was considered statistically significant.

## Results

### Socio-demographic characteristics

The study comprised 822 patients, with 626 from Dhaka Medical College Hospital and 196 from Delta Medical College Hospital. Approximately 60% of the patients sought medical care in the field of medicine, while the remaining 40% required surgical expertise. The largest proportion of patients were from the internal medicine department (42%), followed by pediatrics, general surgery, and Gynecology & Obstetrics (Table 1).

**Table 1:** Socio-demographic characteristics of the patients (*n* = 822)

Characteristic	Total, *n* (%)	Government hospital (*n* = 626), *n* (%)	Private hospital (*n* = 196), *n* (%)	*p*-value
**Specialty category**				
Medical	495 (60.22)	425 (67.89)	70 (35.71)	0.001
Surgical (Surgery and Gynecology & Obstetrics)	327 (39.78)	201 (32.11)	126 (64.29)	
**Specialty**				
Internal Medicine	384 (46.83)	283 (45.35)	59 (30.10)	0.074
Pediatrics	108 (13.17)	98 (15.71)	10 (5.10)	
Gynecology & Obstetrics	83 (10.12)	43 (6.89)	40 (20.41)	
General Surgery	94 (11.46)	40 (6.41)	54 (27.55)	
Orthopedics	52 (6.34)	42 (6.73)	10 (5.10)	
Otolaryngology	49 (5.98)	36 (5.77)	13 (6.63)	
Ophthalmology	49 (5.98)	40 (6.41)	9 (4.59)	
**Department**				
Inpatient	532 (64.72)	400 (63.90)	132 (67.35)	0.485
Outpatient	290 (35.28)	226 (36.10)	64 (32.65)	
**Age of the patient** **(years)**	32.91 (17.88)	31.24 (17.85)	38.24 (16.94)	< 0.001
**Age group (years)**				
< 18	148 (18.00)	130 (20.77)	18 (9.18)	< 0.001
18–30	261 (31.75)	203 (32.43)	58 (29.59)	
31–40	179 (21.78)	137 (21.88)	42 (21.43)	
41–50	98 (11.92)	68 (10.86)	30 (15.31)	
51–60	74 (9.00)	47 (7.51)	27 (13.78)	
> 60	62 (7.54)	41 (6.55)	21 (10.71)	
**Sex**				
Male	513 (62.41)	398 (63.58)	115 (58.67)	0.248
Female	309 (37.59)	228 (36.42)	81 (41.33)	
**Residence**				
Rural	296 (36.01)	296 (47.28)	0 (0.00)	< 0.001
Urban	526 (63.99)	330 (52.72)	196 (100.00)	
**Religion**				
Islam	744 (90.51)	572 (91.37)	172 (87.76)	0.133
Others	78 (9.49)	54 (8.63)	24 (12.24)	
**Educational attainment**				
No formal education	92 (11.19)	81 (12.94)	11 (5.61)	< 0.001
Primary	235 (28.59)	206 (32.91)	29 (14.80)	
Secondary/higher secondary	240 (29.20)	174 (27.80)	66 (33.67)	
University graduate	255 (31.02)	165 (26.36)	90 (45.92)	
**Employment status**				
Employed	413 (50.24)	322 (51.44)	91 (46.43)	0.238
Unemployed	409 (49.76)	304 (48.56)	105 (53.57)	
**Family income**				
Low (< BDT 20,000)	172 (20.92)	144 (23.00)	28 (14.29)	0.039
Middle (BDT 20,000–40,000)	356 (43.31)	282 (45.05)	74 (37.76)	
High (> BDT 40,000)	294 (35.76)	200 (31.95)	94 (47.96)	
Awareness about the referral system*				
No	477 (58.03)	342 (54.63)	135 (68.88)	< 0.001
Yes	345 (41.97)	284 (45.37)	61 (31.12)	
**Distance from the attending hospital**				
< 20 km	323 (39.30)	291 (46.49)	32 (16.33)	< 0.001
> 20 km	499 (60.70)	335 (53.51)	164 (83.67)	

* Awareness about referral system was defined from the question, ‘Have you heard about referral system in healthcare?’

On average, patients were 33 years old (SD 18 years), with nearly two-thirds of them being male. Socio-economically, 43% of the patients were from middle-income households, 21% from low-income backgrounds, and 36% from high-income families (Table 1).

Significant differences were observed in the patient groups from the government and private hospitals with respect to age, residence, educational level, and family income (Table 1).

### Awareness about the referral system

Almost 42% of the patients reported that they were aware about the referral system, which means they knew that an institutional referral is required for admission in the tertiary care hospitals while the remaining 58% of the patients reported they were not aware about the referral system (Table 1).

### Pattern of referral

Out of a total of 822 cases, approximately 59% were self-referred, while 41% were referred by other healthcare facilities, termed institutional referrals (see Fig. 2a). The primary reasons for self-referral included inadequate treatment (28%), inadequate facilities (23%), critical cases (14%), and lack of expert physicians (8%). Conversely, institutional referrals were primarily due to inadequate facilities to treat the patient (53%), inadequate treatment (47%), difficult cases to treat (44%), and lack of expert physicians (31%) at that time. Further details are provided in Fig. 2a.

**Fig. 2:**
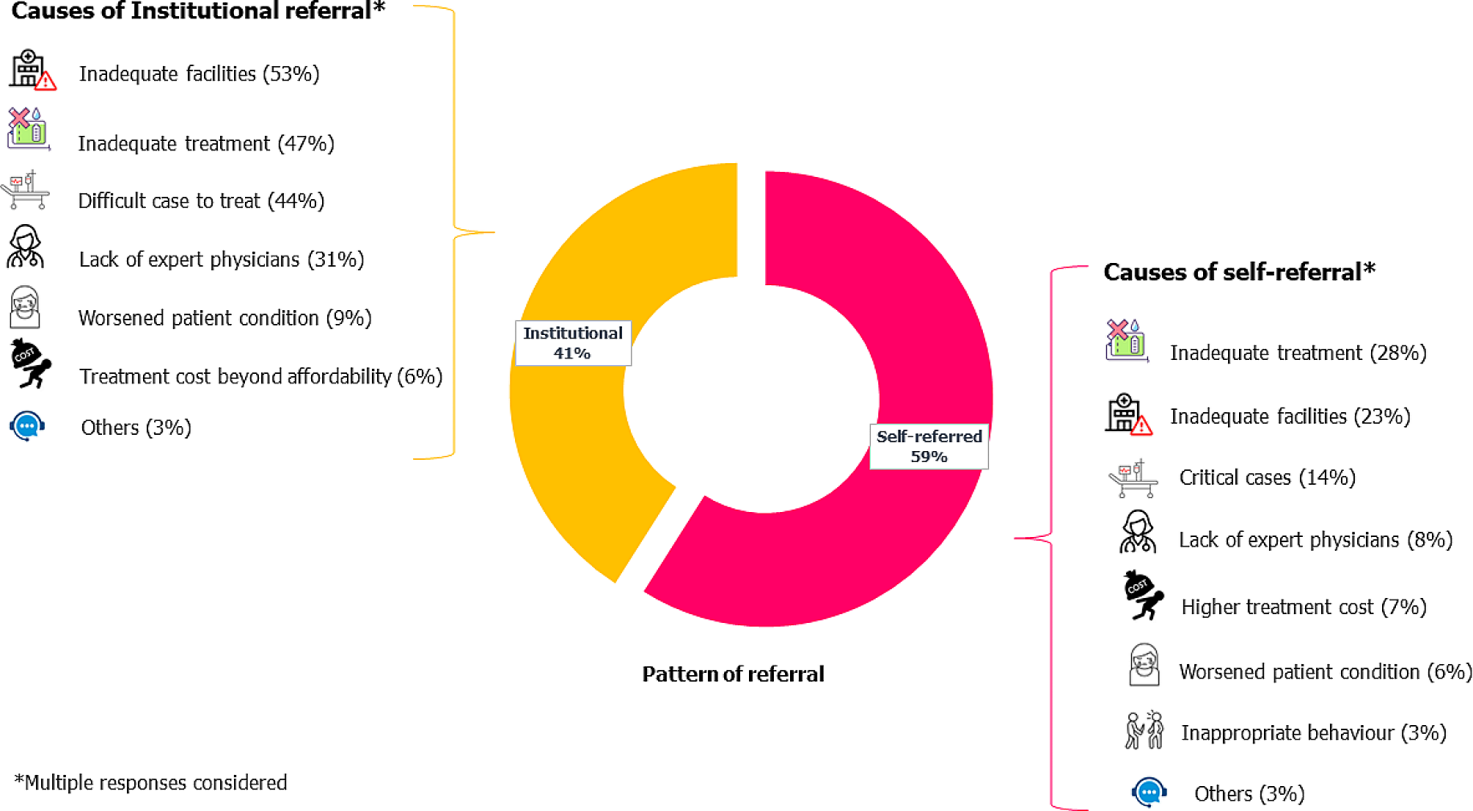
(**a**): Referral patterns and associated causes

**Fig. 2:**
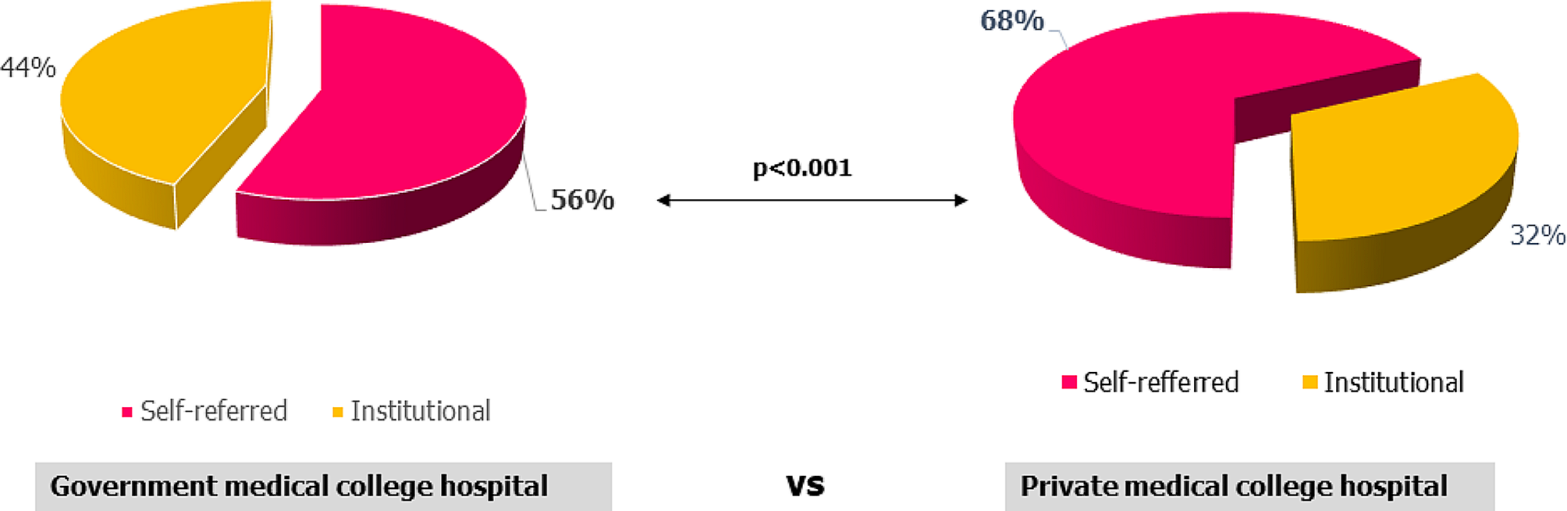
(**b**): Prevalence of self-referral and institutional referral across different institute

In private facilities such as Delta Medical College Hospital, nearly 68% of patients were self-referred, compared to approximately 56% in government facilities like Dhaka Medical College Hospital. The prevalence of self-referral in private hospitals was significantly higher than in government hospitals (*p*-value 0.001), as depicted in Fig. 2b. Further insight into the reasons for referral across different institutions is provided in Table 2.

**Table 2:** Cause of referral and origin of referral across different institutes

Variables	Overall	Govt	Private	*p*-value
**Causes of self-referral according to response of the patient**				
Inadequate treatment	134 (27.80)	93 (26.65)	41 (30.83)	0.3
Inadequate facilities	109 (22.61)	70 (20.05)	39 (29.33)	0.3
Inappropriate behavior	16 (3.32)	8 (2.29)	8 (6.02)	0.050
Higher cost	33 (6.85)	27 (7.74)	6 (4.51)	0.2
Patients condition deterioration	28 (5.81)	14 (4.01)	14 (10.53)	0.006
Critical cases	66 (13.69)	37 (10.61)	29 (21.81)	0.8
Lack of expert physicians	40 (8.30)	24 (6.88)	16 (12.03)	0.067
Others	15 (3.11)	14 (4.01)	1 (0.75)	0.079
**Causes of Institutional referral according to response of the patient**				
Inadequate treatment	160 (47.2)	125 (45.29)	25 (55.56)	< 0.001
Inadequate facilities	180 (53.09)	136 (49.27)	44 (69.83)	< 0.001
Higher cost	22 (6.49)	14 (5.07)	8 (12.70)	0.042
Patients condition deterioration	30 (8.85)	12 (4.35)	18 (28.57)	< 0.001
Difficult cases	150 (44.24)	114 (41.31)	36 (57.15)	0.2
Lack of expert physicians	106 (31.27)	94 (34.06)	12 (19.05)	0.020
Others	20 (5.90)	20 (7.25)	0 (0.00)	0.032
**Origins of Institutional Referrals Received**				
Community clinic	33 (9.73)	16 (5.80)	17 (26.98)	< 0.001
Union sub-center	19 (5.60)	7 (2.54)	12 (19.05)	< 0.001
Upazila Health complex	85 (25.07)	78 (28.26)	7 (11.11)	0.005
District hospital	73 (21.53)	54 (19.57)	19 (30.16)	0.065
Other tertiary hospitals	75 (22.12)	60 (21.74)	15 (23.81)	0.7
Private clinics	142 (41.89)	109 (39.49)	33 (52.38)	0.061

### Origins of institutional referral

Among these patients, nearly 10% were referred from Community Clinics, 6% from Union Sub-Centers, 25% from Upazila Health Complexes, 22% from District Hospitals, 22% from other tertiary care hospitals, and 42% from private clinics. Table 2 provides a breakdown of the origin of referrals in both government and private institutions.

### Factors associated with self-referral

Our logistic regression analysis revealed several factors significantly associated with an increased likelihood of patients self-referring to tertiary care facilities. These factors included visiting the outpatient department (adjusted odds ratio [aOR] 3.3, 95% CI 2.28–4.82, *p*-value < 0.001), residing in urban areas (aOR 1.29, 95% CI 1.04–1.64, *p*-value 0.007), belonging to middle- and high-income families (aOR 1.34, 95% CI 1.03–1.62, *p*-value 0.014, and aOR 1.98, 95% CI 1.54–2.46, *p*-value 0.005 respectively), and living within a 20 km radius of healthcare facilities (aOR 3.15, 95% CI 2.24–4.44, *p*-value < 0.001) (Table 3).

**Table 3:** Factors associated with self-referral of the patients (logistic regression model)

Characteristic	Yes	No	cOR (95% CI)	*p*-value	aOR (95% CI)	*p*-value
Hospital type						
Government	350 (55.91)	276 (44.09)				
Private	133 (67.86)	63 (32.14)	1.66 (1.19, 2.35)	0.003	1.49 (0.89, 2.50)	0.13
**Specialty category**						
Medical	303 (61.21)	192 (38.79)				
Surgical	180 (55.05)	147 (44.95)	0.78 (0.58, 1.03)	0.079		
**Department**						
Inpatient	255 (47.93)	277 (52.07)				
Outpatient	228 (78.62)	62 (21.38)	3.99 (2.89, 5.58)	< 0.001	3.3 (2.28, 4.82)	< 0.001
**Sex**						
Male	300 (58.48)	213 (41.52)				
Female	183 (59.22)	126 (40.78)	1.03 (0.77, 1.37)	0.8		
**Age group**						
< 18	88 (59.46)	60 (40.54)				
18–30	158 (60.54)	103 (39.46)	1.05 (0.69, 1.58)	0.8		
31–40	109 (60.89)	70 (39.11)	1.06 (0.68, 1.66)	0.8		
41–50	50 (51.02)	48 (48.98)	0.71 (0.42, 1.19)	0.2		
51–60	47 (63.51)	27 (36.49)	1.19 (0.67, 2.13)	0.6		
> 60	31 (50.00)	31 (50.00)	0.68 (0.37, 1.24)	0.2		
**Residence**						
Rural	151 (51.01)	145 (48.99)				
Urban	332 (63.12)	194 (36.88)	1.64 (1.23, 2.19)	< 0.001	1.29 (1.04, 1.64)	0.007
**Religion**						
Islam	436 (58.60)	308 (41.40)				
Others	47 (60.26)	31 (39.74)	1.07 (0.67, 1.74)	0.8		
**Education**						
No formal education	57 (61.96)	35 (38.04)				
Primary	124 (52.77)	111 (47.23)	0.69 (0.42, 1.12)	0.13		
Secondary/higher secondary	139 (57.92)	101 (42.08)	0.85 (0.51, 1.38)	0.5		
University graduate	163 (63.92)	92 (36.08)	1.09 (0.66, 1.77)	0.7		
**Employment**						
Employed	232 (56.17)	181 (43.83)				
Unemployed	251 (61.37)	158 (38.63)	1.24 (0.94, 1.64)	0.13		
**Family income**						
Low	82 (47.67)	90 (52.33)				
Middle	208 (58.42)	148 (41.57)	1.54 (1.12, 1.98)	0.012	1.34 (1.03, 1.62)	0.014
High	193 (65.64)	101 (34.35)	2.01 (1.66, 2.46)	0.008	1.98 (1.54, 2.46)	0.005
**Distance from the center**						
> 20 km	143 (28.66)	356 (71.34)				
< 20 km	196 (60.68)	127 (39.32)	3.84 (2.86, 5.18)	< 0.001	3.15 (2.24, 4.44)	< 0.001
**Awareness about referral system**						
No	199 (41.72)	278 (58.28)				
Yes	140 (40.58)	205 (59.42)	1.05 (0.79, 1.39)	0.7		

cOR: Crude ODDs ratio, CI: Confidence Interval, aOR: Adjusted ODDs rati

## Discussion

Our research discovered that nearly 59% of patients in the studied tertiary care hospitals opted for self-referral. Notably, individuals seeking outpatient services, urban residents from more affluent backgrounds, and those residing closer to the hospitals demonstrated a higher tendency toward self-referral to tertiary care hospitals. Moreover, patients perceiving inadequacies in primary care facilities were notably inclined to visit tertiary care hospitals directly.

Despite established policies concerning the patient referral system in Bangladesh, implementing these policies into practical application presents considerable challenges. The tertiary care facilities in Bangladesh grapple with a substantial patient load, while primary care centers often remain underutilized [8]. There is a lack of prior evidence on this topic from Bangladesh for comparative analysis. However, our findings showcase a higher self-referral proportion compared to neighboring India, where recent studies reported self-referral rates of approximately 40% [23]. Interestingly, our findings align with similar trends observed in other LMICs, such as Ethiopia (63%) [24] and Nigeria (60%) [25].

The primary reason for patients self-referring in our study was their perception of inadequate treatment and diagnostic facilities at primary care centers. Similarly, patients referred by primary or secondary care facilities cited similar issues, such as a lack of expert physicians and appropriate diagnostic and management resources in the referring hospitals. Many of our study participants believed there was a scarcity of healthcare facilities in primary care centers, which strongly correlated with patients’ decisions to self-refer. This deficiency in staff and healthcare amenities within primary care settings is not unique to Bangladesh but prevails in many developing countries [26–28]. These shortcomings drive patients to bypass primary health facilities, mirroring observations in other developing countries like Ethiopia and Nigeria, where patients preferred self-referral to secondary or tertiary care facilities due to their negative perceptions about the care providers and lack of adequate treatment facilities [7, 24, 29, 30].

Our research further revealed that a significant number of patients self-referred to tertiary care facilities due to suggestions from relatives or friends when facing health issues. Hence, the inclination to skip primary care facilities for higher-level care often stems from external encouragement, such as advice from participants’ families and friends, consistent with findings from prior studies [7, 31, 32]. Moreover, over half of our participants were unaware of the referral system, potentially influencing their decisions to seek healthcare at tertiary levels. Studies have indicated that patients unaware of the referral system are more likely to self-refer [24, 25, 33]. Increasing awareness about the referral system could enhance patients’ understanding of the overall service provision across healthcare facilities and the connection between lower-tier and higher-tier healthcare establishments [34].

Various socio-demographic factors were found to be linked with self-referral among our participants. Patients residing in urban areas and belonging to middle- and high-income families displayed a higher tendency to self-refer to tertiary care facilities. Additionally, individuals visiting the outpatient department were more likely to seek care directly from tertiary care hospitals. Although we observed a higher rate of self-referral in private hospitals compared to government hospitals, this difference did not hold statistical significance upon further analysis. Most of these factors are intertwined with the participants’ socioeconomic status, a phenomenon observed in studies conducted across different countries [24, 35]. Notably, individuals from middle- and higher-income families possess greater financial capability for medical expenses and are more aware that higher-level health facilities offer better healthcare, potentially influencing their choice to seek medical care from such centers [24]. Finally, our study indicated that patients living closer to tertiary care hospitals were more likely to bypass primary care centers. Various studies from different countries have reported that longer travel times to primary facilities compared to higher-tier facilities increase the likelihood of self-referral [24, 36, 37].

This study offers insights into the patterns and prevalence of self-referral among patients in selected tertiary care hospitals in Bangladesh. The findings underscore the need for an evaluation of the current healthcare referral system model, particularly in resource-constrained settings like Bangladesh, where primary care centers often lack necessary resources [26].

Several limitations of this study should be noted. Firstly, the research was conducted solely among patients visiting two specific tertiary care hospitals located in Dhaka city. Additionally, we included the patients from specific subspecialties of only two tertiary care hospitals, which might not depict the overall picture of the country. Moreover, there are methodological limitations, such as selection bias. Despite our systematic randomized sampling approach, there remains a potential risk of selection bias because there could be a non-random difference between self-referred patients and those referred by institutions, which might have influenced their decision to participate in this study. Besides, the questionnaire used in this study was not validated in the context of Bangladesh, which was also a significant limitation. Finally, the sample size was disproportionate in government and private hospitals, which might have biased the findings. Moreover, though our calculated sample size was 864, we could include 822 patients in the final analysis, which might underpower our statistical analysis. As a quantitative study, this research primarily focused on identifying specific factors influencing patients’ self-referral. Therefore, future qualitative studies could provide a more in-depth understanding of patients’ self-referral behavior.

## Conclusion

Our study highlights a significant trend where a considerable number of patients accessing tertiary care facilities in Bangladesh choose to bypass the institutional referral system. The most frequently cited reason for self-referral among these patients is the perceived inadequacy of facilities in primary care centers. Furthermore, individuals from affluent backgrounds in urban areas and those residing in close proximity to tertiary care facilities are more prone to self-refer. This inclination towards self-referral might strain the limited resources available at tertiary care facilities. Urgent measures are required to establish an effective referral system and bolster the capacity of primary care centers. These actions are essential to enhance the efficiency of Bangladesh’s healthcare system.

### Electronic supplementary material

Below is the link to the electronic supplementary material.

Supplementary Material 1

Supplementary Material 2

## Data Availability

Patient-level data will be available at the request of the corresponding author.

## Abbreviations

CI: Confidence Interval
ERC: Ethical Review Committee
HDU: High Dependency Unit
ICU: Intensive Care Unit
LMICs: Low-And Middle-Income Countries
OR: Odds Ratios
SD: Standard Deviation
