# Supplementary material for: Patient self-referral patterns in a developing country: characteristics, prevalence, and predictors
Source: BMC Health Serv Res. 2024 May 21;24:651. doi: 10.1186/s12913-024-11115-8 (PMC11110194; doi:10.1186/s12913-024-11115-8)
Supplement: Supplementary file 1 — Supplementary Material 1 [file 12913_2024_11115_MOESM1_ESM.docx]

**Understanding the health system in Bangladesh:**

Bangladesh has a comprehensive health infrastructure that spans across the country. The country has eight administrative divisions and 64 districts, and the districts are divided into upazilas . These upazilas, in turn, are composed of unions, and each union is subdivided into nine wards, with each ward encompassing one or more villages. The hierarchical structure of Bangladesh's health system resembles a multi-layered pyramid. More details are illustrated in **Figure 1.**

**At the village-level Satellite clinic:**

At the village level, Satellite Clinics (SC) represent a pivotal outreach initiative established by the Government of Bangladesh in 1984 [10]. Typically, these outreach centers provide services to 3000-4000 people at the village (ward consists of one or more villages) level. The primary objective was to extend health and family planning services to rural populations who reside in remote areas far away from medical facilities. These clinics strategically operate in hard-to-reach locations and areas distant from fixed centers, such as community clinics or other government health settings. According to operational guidelines, these clinics are scheduled to operate the clinic in eight times in eight different wards in a month from 9 am to 2.30 pm. Apart from general health services, the clinics actively contribute to the Expanded Program on Immunization (EPI), commonly recognized at the community level as a vaccination center [11].

**At ward-level Community clinic:**

Community clinic serves as the base of the fixed health infrastructure in Bangladesh health system, strategically located in the ward level and designed to provide care to 6,000-8,000 population [12]. It functions as a comprehensive 'one-stop' service outlet, providing a spectrum of services encompassing health, family planning, and nutrition, with an emphasis on preventive care and health promotion [12,13,14,15].

This health facility is staffed with a team comprising a health assistant, a family welfare assistant, and Community Health Care Providers (CHCP). CHCP works 6 days a week, ensuring consistent availability for the community. Additionally, the health assistant (HA) and family welfare assistant (FWA) operate on an alternate schedule, each contributing three days a week to provide a well-rounded and comprehensive healthcare service throughout the week [14, 15].

**Health facilities at the union level:**

At the union level, three types of health facilities play a crucial role in providing healthcare services. They are- i) Union Health and Family Welfare Centre (UHFWC), ii) the union sub-center, and iii) rural health centers. These centers provide outpatient care for every 24,000 to 30,000 population. To ensure the delivery of essential health services, all union facilities are staffed with Sub-Assistant Community Medical Officers (SACMO) who play a pivotal role in providing healthcare services to the local population. Additionally, the Family Planning Department, through UHFWCs, offers Maternal and Child Health (MCH) and Family Planning (FP) services, which are staffed with qualified paramedics [16].

**Health facilities at sub-district level-Upazila health complexes:**

At the sub-district level, the health department provides preventive, promotive, and curative services through Upazila health complexes (UHCs) for every 250,000 population. The in-patient capacity of UHCs ranges between 31 to 50 beds. In addition to primary care services, some UHCs also offer secondary care, including comprehensive emergency obstetrical care (EOC). In most of the UHC buildings, the family planning department also has a separate unit of maternal and child health (MCH) care services at the sub-district level physically located mostly in the same building of UHC, streamlining access to critical reproductive and child health services. In addition to in-patient care, all UHCs also provide treatment on an OPD basis. Limited surgeries were also conducted at the upazila level, especially in 50-bedded UHCs. Despite various shortcomings, UHCs are considered the first referral level of the health system [16].

**Secondary care hospitals**:

District hospitals (DHs) offer a secondary level of healthcare to a wide range of the population in Bangladesh, serving as second-referral centers that receive referrals from primary-level health facilities. At the district level, the Health Department ensures secondary care for approximately 2,500,000 people through district or general hospitals, varying in bed capacity from 100 to 250. Some hospitals in certain districts are referred to as 'general hospitals' or '250-bed hospitals’ [17]. They provide both primary and secondary care through outdoor and inpatient departments, encompassing outpatient and inpatient services, as well as emergency care. In some districts, there is a medical college with hospitals that offer tertiary care alongside primary and secondary services. In addition, there are other types of health facilities at the district level, such as the Maternal and Child Welfare Center (MCWC), that provide care for maternal and child health and FP services. They also provide emergency obstetrical care. The usual capacity of these hospitals ranges from 10-20 beds [17].

**Tertiary care hospitals:**

Tertiary care facilities are advanced healthcare institutions that offer specialized medical services that are not present in primary and secondary care institutions, and they are equipped with various disciplines and advanced laboratory facilities. Usually, experienced and senior staff in all ranges, including doctors, nurses, and other health care staff, provide care to the patient. At the national level, these facilities may include super-specialty hospitals providing high-end medical services in specific fields. The Health Department in Bangladesh administers tertiary-level services through teaching hospitals and specialized hospitals located in divisional headquarters [18]. On a national scale, there are institutions dedicated to both pre-service education and postgraduate medical education/training, coupled with specialized treatment for patients. Most of the specialized institutes focusing on cardiovascular, traumatology, kidney, mental health, cancer, neuroscience, ear-nose-throat, ophthalmology, chest diseases, and more are situated in Dhaka, the capital of Bangladesh. The highest capacity of these tertiary care centers is up to 2400 beds. Not only do they provide in-patient care, but they also provide care to patients on an outpatient basis and through emergency departments. In addition, there are two 100-bed maternal and child health hospitals and infertility clinics run by the family planning department. Both of the hospitals are also located in Dhaka [16,18].

**Other health settings:**

In addition to the regular public health care network, there are additional health facilities run by different organs of the government, e.g. the Combined Military Hospital (CMH) operated under the Ministry of Defence provides care to the defense force; Police hospital, which is operated by the Ministry of Home Affairs, Railway hospitals by the Ministry of Railways, and Jail hospital by the Ministry of Home Affairs, Institute of Nuclear Medicine and Allied Sciences, under the Ministry of Science & Technology, etc. [16]. Furthermore, primary healthcare services in urban areas are under the administration of the Ministry of Local Government, Rural Development, and Cooperatives (MOLGRD&C), significantly contributing to the well-being of the broader community within their respective catchment areas [16].
