# Supplementary material for: Patient self-referral patterns in a developing country: characteristics, prevalence, and predictors
Source: BMC Health Serv Res. 2024 May 21;24:651. doi: 10.1186/s12913-024-11115-8 (PMC11110194; doi:10.1186/s12913-024-11115-8)
Supplement: Supplementary file 2 — Supplementary Material 2 [file 12913_2024_11115_MOESM2_ESM.docx]

**(Questionnaire-English)**

**Title: “Patient Self-Referral Patterns in a Developing Country: characteristics, prevalence, and predictors”**

I would like to start by asking you some background questions before asking you questions on your health. This information is confidential and will only be used for research purposes.

| **Question no.** | **Questions** | **Possible response with instructions** | |
| --- | --- | --- | --- |
| Q.01 | What is the name of the Hospital? | **______________________** | |
|  | Name of the Department |  | |
|  |  | **In-patient** | **Outpatient** |
| Q.02 | Record Sex as Observed | Male | Female |
| Q.03 | How old are you? |  | |
| Q.04 | What is the address of Resident? | Village/area of house:……………………..  Thana/Upazila  Municipality/city corporation  District: | |
| Q.05 | What is the Religion? | 1. Islam | |
|  |  | 2. Hindu | |
|  |  | 3. Christian | |
|  |  | 4. Buddhist | |
|  |  | 5. Others (please specify) ……………. | |
| Q.06 | What is your current job? | 1. Government Employee | |
|  |  | 2. Non-Government employee | |
|  |  | 3. Self-employed | |
|  |  | 4. Employer | |
|  |  | 5. Not working for pay (If not working for pay: please answer Q.208) | |
| Q.07 | During the last 12 months, what has been your main occupation? | 1. Legislator, Senior Official, or Manager | |
|  |  | 2. Professional (engineer, doctor, teacher, clergy, etc.) | |
|  |  | 3. Technician or Associate Professional (inspector, finance dealer, etc.) | |
|  |  | 4. Clerk (secretary, cashier, etc.) | |
|  |  | 5. Service or sales worker (cook, travel guide, shop salesperson, etc.) | |
|  |  | 6. Agricultural or fishery worker (vegetable grower, livestock producer, etc.) | |
|  |  | 7. Craft or trades worker (carpenter, painter, jewelry worker, butcher, etc.) | |
|  |  | 8. Plant/machine operator or assembler (equipment assembler, sewing-machine operator, driver, etc.) | |
|  |  | 9. Elementary worker (street food vendor, shoe cleaner, etc.) | |
|  |  | 10. Armed forces (government military) | |
| Q.08 | What is the main reason you are not working for pay? | 1. Homemaker / caring for family | |
|  |  | 2. Looked but can’t find a job | |
|  |  | 3. Doing unpaid work / voluntary activities | |
|  |  | 4. Studies / training | |
|  |  | 5. Retired / too old to work | |
|  |  | 6. Ill health | |
|  |  | 7. (please specify) ……………. | |
| Q.09 | What is the highest level of education that you have completed? | 1. No formal schooling | |
|  |  | 2. Less than primary school | |
|  |  | 3. Primary school completed | |
|  |  | 4. Secondary school completed | |
|  |  | 5. High school (or equivalent) completed | |
|  |  | 6. College / pre-university / University completed | |
|  |  | 7. Post graduate degree completed | |
| Q.10 | Diagnosis |  | |
|  | **Utilization of referral system (Source of referral)** | | |
| Q.11 | Who advise you to come here (admit this hospital)?/OPD visit? | 1.Self: a.Yes b.No; If yes, please answer **Q 212 and If No (Q. no:2-12), please answer Q 213**  2. Kabiraz: a.Yes b.No  3. Palli doctor: a.Yes b.No  4. Friends or family: a.Yes b.No  5. Village doctor: a.Yes b.No  6. community clinic: a.Yes b.No  7. Union subcenter: a.Yes b.No  8. Upazilla Health complex: a.Yes b.No  9. District hospital: a.Yes b.No  10. Another tertiary hospital: a.Yes b.No; if yes, please specify……………………  11. Private clinic: a.Yes b.No  12.Others (please specify) …………….  **Please try to make a flow chart about the referral centers used by patients:**  ……………………………… | |
| Q.12 | Reasons for referring patients by self: | **Response or comments done by attendant or care giver or by patients himself: (More than one response could be possible)**  **1.** Inadequate treatment: a.Yes b.No; If yes, please specify  **2.** Inadequate facilities: a.Yes b.No; If yes, please specify  **3.** Inappropriate behavior: a.Yes b.No; If yes, please specify  **4.** Higher cost: a.Yes b.No; If yes, please specify  **5.** Patients condition deteriorating: a.Yes b.No; If yes, please specify  **6.** Others: : a.Yes b.No; If yes, please specify……………………………… | |
| Q.13 | Reasons for referring patients by hospital facilities: | **Response or comments done by physicians: (More than one response could be possible)**  **1.** Difficult cases: a.Yes b.No; If yes, please specify  **2.** Required additional management (eg chemotherapy etc) : a.Yes b.No; If yes, please specify  **3.** Required additional equipment for diagnosis (eg CT scan, MRI, angiogram etc) : a.Yes b.No; If yes, please specify  **4.** Lack of expert physicians (eg. anesthetists for CS): a.Yes b.No; If yes, please specify  **5.** Lack of appropriate facility (eg. ICU etc) : a.Yes b.No; If yes, please specify  **6.** When the illness has no solution (end stage disease) : a.Yes b.No; If yes, please specify  7. Others: : a.Yes b.No; If yes, please specify………………………………………. | |
| **Knowledge and perception of referral system of health care service** | | | |
| Q.14 | If hospital facilities referred the patients, have the patient received referral note? | 1. a.Yes b.No; If **No,** please specify………………………………………. | |
| Q.15 | Do you think, it was a good decision to admit here?? | 1. a.Yes b.No; If yes, please specify………………………………………. | |
| Q.16 | Do you think your doctor rightly suggest you about referral? (if hospital facilities referred the patient) | 1. a.Yes b.No; If **No,** please specify………………………………………. | |
| Q.17 | Do you know when a patient become sick, which one is the first centers you should go? | (Please write the response of the patients)  …………………………………………….. | |
| Q.18 | Do you know, before going to any higher center, a referral note is required? | (Please write the response of the patients)  …………………………………………….. | |
| Q.19 | Do you know any existing referral system in Bangladesh? | (Please write the response of the patients) | |
| Q.20 | Do you know, without a referral note, any tertiary care center can refuse your patients? | (Please write the response of the patients) | |
| Q.21 | Do you know, without a referral note, any tertiary care center can send back to you lower center? | (Please write the response of the patients) | |
| Q.22 | Distance of the hospital (km) |  | |
